# Draft Genome Sequence of *Pannonibacter phragmitetus* Strain CGMCC9175, a Halotolerant Polycyclic Aromatic Hydrocarbon-Degrading Bacterium

**DOI:** 10.1128/genomeA.01675-15

**Published:** 2016-01-28

**Authors:** Xinxin Wang, Decai Jin, Lisha Zhou, Zhuo Zhang

**Affiliations:** a School of Environmental Science and Engineering, Tianjin University, Tianjin, China; b Offshore Environmental Service Co. Ltd., Tianjin, China; c Research Center for Eco-Environmental Sciences, Chinese Academy of Sciences, Beijing, China; d Shenzhen Key Laboratory of Environmental Microbial Genomics and Application, BGI, Shenzhen, China; e Hunan Plant Protection Institute, Changsha, China

## Abstract

*Pannonibacter phragmitetus* CGMCC9175 is a halotolerant polycyclic aromatic hydrocarbon (PAH)-degrading bacterium isolated from PAH-contaminated intertidal zone sediment. Here, we report the 5.7-Mb draft genome sequence of this strain, which will provide insights into the diversity of *Pannonibacter* and the mechanism of PAH degradation in sediments.

## GENOME ANNOUNCEMENT

Polycyclic aromatic hydrocarbon (PAH)-degrading bacteria play a key role in the natural remediation of PAH-polluted marine sediments (1), which attract attention from researchers in the field of environmental microbiology. *Pannonibacter phragmitetus* CGMCC9175 was isolated from PAH-contaminated intertidal zone sediment of Bohai Sea in China (2), which can degrade naphthalene, phenanthrene, and anthracene with 5% NaCl. Until recently, only the genome of *P. phragmitetus* DSM 14782 has been available. Here, the draft genome sequence of *P. phragmitetus* CGMCC9175 is presented.

Genomic DNA was extracted and sequenced using the Illumina MiSeq platform. The shotgun sequencing produced 5,361,830 paired-end reads, with an average insert size of 300 bp, yielding approximately 280-fold coverage; the reads were filtered by the NGS QC toolkit version 2.3 (3). The filtered reads were assembled, scaffolded, gap filled, and validated using SOAP*denovo* version 2.04 (4), SSPACE version 2.0 (5), GapFiller version 1.10 (6), and bwa version 0.7.4 (7). The final assembly consisted of 40 contigs, with an *N*_50_ length of 353,668 bp and a largest length of 653,707 bp. The genome sequence was annotated using the NCBI Prokaryotic Genome Annotation Pipeline (PGAP) (http://www.ncbi.nlm.nih.gov/genome/annotation_prok/).

The genome consists of 5.7 Mb, with a G+C content of 63.6%. A total of 4,853 coding sequences (CDSs), 167 pseudogenes, 50 tRNAs, 1 noncoding RNA (ncRNA), and 1 rRNA operon were identified. Of the CDSs, 86.1% can be assigned to COGs, with amino acid transport and metabolism as the most abundant class, and 44.1% can be annotated into 2,286 KEGG orthologous groups using KAAS (8), involving 233 metabolic pathways. The ISAs1 family dominated the insertion sequence (IS) elements, as revealed by ISFinder (9). Plasmid-carried genes essential for stabilization and partition were detected, which suggests the presence of plasmid. A total of 199 tandem repeats, 370 potentially secreted proteins, 3 prophage sequences, and 6 clustered regularly interspaced short palindromic repeat (CRISPR) elements were identified by Tandem Repeats Finder version 4.08 (10), SignalP version 4.1 (11), PHAST (12), and CRISPRFinder (13), respectively. Average nucleotide identity (ANI) analysis (14) revealed that *P. phragmitetus* CGMCC9175 is phylogenetically related to *P. phragmitetus* DSM 14782 (93.3%).

One alkane 1-monooxygenase, 1 catechol 1,2-dioxygenase, 1 homogentisate 1,2-dioxygenase, 2 protocatechuate 3,4-dioxygenase, and 2 biphenyl 2,3-dioxygenase genes were identified, which were responsible for the degradation of alkanes and PAHs. One 2-haloalkanoic acid dehalogenase and 6 haloacid dehalogenase genes were identified, which were responsible for the degradation of halogenated organic compounds. Moreover, 3 genes were identified as being involved in compatible solute synthesis and uptake, including 1 glycine/betaine ABC transporter and 2 betaine-aldehyde dehydrogenase. These genes may be essential to the survival in a hydrocarbon-contaminated saline environment. Information about the genome sequence of *P. phragmitetus* CGMCC9175 offered an opportunity to understand the genetic diversity of *Pannonibacter* and the mechanism of hydrocarbon degradation in intertidal zone sediments.

### Nucleotide sequence accession number.

The draft genome sequence of *P. phragmitetus* CGMCC9175 has been deposited in GenBank under the accession number LGSQ00000000. The version described in this paper is the first version.
